# Characterizing Geosocial-Networking App Use Among Young Black Men Who Have Sex With Men: A Multi-City Cross-Sectional Survey in the Southern United States

**DOI:** 10.2196/10316

**Published:** 2018-06-14

**Authors:** Dustin T Duncan, Su Hyun Park, H Rhodes Hambrick, Derek T Dangerfield II, William C Goedel, Russell Brewer, Ofole Mgbako, Joseph Lindsey, Seann D Regan, DeMarc A Hickson

**Affiliations:** ^1^ Spatial Epidemiology Lab Department of Population Health New York University School of Medicine New York, NY United States; ^2^ The REACH Initiative Johns Hopkins University School of Nursing Baltimore, MD United States; ^3^ Chicago Center for HIV Elimination Department of Medicine University of Chicago Chicago, IL United States; ^4^ MBK Gulf Coast My Brother's Keeper, Inc Gulfport, MS United States; ^5^ Us Helping Us, People Into Living, Inc Washington, DC United States

**Keywords:** HIV prevention, black MSM, dating apps, homosexuality, male, men who have sex with men, mobile apps, mobile phones

## Abstract

**Background:**

Understanding where and how young black men who have sex with men (YBMSM) in the southern United States meet their sexual partners is germane to understanding the underlying factors contributing to the ongoing HIV transmission in this community. Men who have sex with men (MSM) commonly use geosocial networking apps to meet sexual partners. However, there is a lack of literature exploring geosocial networking app use in this particular population.

**Objective:**

Our aim was to examine the characteristics, preferences, and behaviors of a geographically diverse sample of geosocial networking app-using YBMSM in the southern United States.

**Methods:**

Data were collected from a sample of 75 YBMSM across three cities (Gulfport, Mississippi; Jackson, Mississippi; and New Orleans, Louisiana). Multiple aspects of geosocial networking app use were assessed, including overall app use, age of participant at first app use, specific apps used, reasons for app use, photos presented on apps, logon times and duration, number of messages sent and received, and characteristics of and behaviors with partners met on apps. Survey measures of app-met partner and sexual behavior characteristics assessed at midpoint (Day 7) and completion visits (Day 14) were compared using McNemar’s test or Wilcoxon signed-rank test. In addition, we assessed activity spaces derived from GPS devices that participants wore for 2 weeks.

**Results:**

Of the 70 participants who responded to the overall app-use item, almost three-quarters (53/70, 76%) had ever used geosocial networking apps. Jack’d was the most commonly used geosocial networking app (37/53, 70%), followed by Adam4Adam (22/53, 42%), and Grindr (19/53, 36%). The mean and median number of apps used were 4.3 (SD 2.7) and 4.0 (range 0-13), respectively. Most app-using participants displayed their face on the profile picture (35/52, 67%), whereas fewer displayed their bare legs (2/52, 4%) or bare buttocks (or ass; 2/52, 4%). The mean age at the initiation of app use was 20.1 years (SD 2.78) ranging from 13-26 years. Two-thirds (35/53, 66%) of the sample reported using the apps to “kill time” when bored. A minority (9/53, 17%) reported using the apps to meet people to have sex/hook up with. The vast majority of participants reported meeting black partners for sex. Over two-thirds (36/53, 68%) reported that the HIV status of their app-met partners was negative, and 26% (14/53) reported that they did not know their partner’s HIV status. There was a significant difference in GPS activity spaces between app using YBMSM compared to nonapp using YBMSM (2719.54 km^2^ vs 1855.68 km^2^, *P*=.011).

**Conclusions:**

Use of geosocial networking apps to meet sexual partners among our sample of YBMSM in the southern United States was common, with a diverse range of app use behaviors being reported. Further research should characterize the association between geosocial networking app use and engagement in sexual behaviors that increase risk for HIV acquisition and transmission. In addition, geosocial networking apps present a promising platform for HIV prevention interventions targeting YBMSM who use these apps.

## Introduction

While overall HIV incidence in the United States has declined over the past several years [1], this progress has not been shared equally among all demographic subgroups or geographic regions. Black men who have sex with men (BMSM) continue to account for the greatest proportion of new HIV diagnoses of any demographic group, despite reporting higher engagement in prevention behaviors such as condom use and fewer sexual partners compared to other groups of men who have sex with men (MSM) [2,3]. Estimated lifetime risk for HIV infection for BMSM is 50% [4], and despite a potentially large benefit from pre-exposure prophylaxis (PrEP) use [5], the awareness, access, and use of PrEP are low among BMSM [6-8]. Young BMSM (YBMSM) in the “Deep South,” the geographic region in the southern United States comprising Georgia, Alabama, Arkansas, South Carolina, Mississippi, and Louisiana, are an especially high-priority community for HIV prevention efforts. Of all new HIV diagnoses among BMSM in 2016, 75% occurred among those aged 13-29 years [3], and more than half occurred in the Deep South [3].

Millions of MSM worldwide use geosocial networking (GSN) apps to meet romantic and sexual partners [9-11], and approximately 33% of MSM have used GSN apps in their lifetime [11]. Up to 85% of app-using MSM engage with apps daily [12]. Given the increasing integration of mobile phone use into daily living activities, current trends in GSN app use by MSM will likely continue, if not increase, in coming years. The importance of mobile phones in daily life warrants further investigation of GSN app use, beliefs, and behaviors in priority populations such as YBMSM in the Deep South, the epicenter of the HIV epidemic in the United States.

The available evidence affirms that app-using BMSM constitute a high-priority population for HIV prevention. Therefore, understanding where and how YBMSM in the Deep South meet their sexual partners is germane to understanding the underlying factors contributing to their HIV risk. The role of GSN apps in the life of YBMSM in the Deep South is especially important to study because unlike in urban areas, there are few (if any) venues available for socializing or developing relationships, so GSN apps could play a more significant role in these processes. The Deep South is also different from regions where research has typically been conducted because of its long-standing history and continued instances of oppression, racism, and homophobia (eg, legislation that will allow for businesses to not serve or provide services to lesbian, gay, and bisexual individuals based on their religious beliefs).

The literature shows mixed evidence for the association between GSN app use and sexual risk among MSM. Some studies have found that app-using MSM commonly engage in sexual behaviors associated with an elevated risk of HIV infection [13-15] compared to MSM who did not use apps. Such behaviors include having multiple sexual partners, engaging in condomless anal intercourse (CAI), engaging in group sex, and using alcohol and drugs during sex [11,14-22]. App-using MSM have also been shown to have a higher prevalence of sexually transmitted infection diagnosis, which is associated with an elevated risk of HIV seroconversion [11,14,23]. However, other studies show similar rates of sexual risk behaviors between MSM who use GSN apps and those who do not [17,24]. Interestingly, a study of BMSM in 6 cities found that HIV-uninfected participants were more likely to engage in serodiscordant / serostatus-unknown CAI with partners met offline at sex-focused venues than with partners met online [25]. A recent meta-analysis of app-using MSM found that 46% of MSM had CAI with all partners within the previous 3 months, yet as many as 70% of those engaging in CAI had a low self-perceived risk of HIV [11]. Despite these mixed findings, GSN apps constitute a potential platform for dissemination of HIV prevention-related information or interventions [26], such as reminders for HIV testing or delivery of home HIV test kits [27,28], identifying locations for HIV testing and treatment centers [12,18,29], and recruitment for inclusion in studies of novel HIV prevention and treatment methods [16,30].

Maximizing the potential efficacy of interventions will necessitate a rich understanding of the personal characteristics, preferences, and behaviors of app-using MSM subgroups, such that interventions will be socioculturally informed and acceptable to the populations they are designed to benefit. There is a lack of literature available on app-using YBMSM in most app-based studies. Even in studies based in the Deep South, study populations are multiracial or have samples of predominantly white MSM, thereby limiting their generalizability to BMSM. A study by Goedel and Duncan of GSN app use among 92 MSM in Atlanta, Georgia, for instance, included only 19% black participants [9]. A study of 110 MSM in Washington, DC, by Lehmiller et al included only 14% nonwhite participants [14]. Given the lack of literature specifically exploring GSN app use in this population, we conducted a survey of the characteristics, preferences, and behaviors of a geographically diverse sample of GSN app-using black MSM in the Deep South. We evaluated a range of GSN apps, including Grindr, Jack’d, and Hornet. Because YBMSM may also use more mainstream social medial platforms such as Instagram to find sex partners, we included these platforms in our analysis as well.

## Methods

### Study Design

This study was primarily designed to understand the feasibility of collecting global positioning system (GPS) data over 2 weeks from BMSM in four cities in the Deep South: Gulfport, Mississippi; Hattiesburg, Mississippi; Jackson, Mississippi; and New Orleans, Louisiana. These locations were selected because they are locations where we had community partners. Of note, Jackson leads the nation with the number of MSM living with HIV (4 in 10) [31], and surveillance data suggest that 3 in 4 of these men are black [32]. Both Jackson and New Orleans are in the top 5 cities in the nation for the number/rate of new infections [3]. Although reported anecdotally, there are major thoroughfares that connect all four of these cities (eg, Interstate 10 and US Highway 29), and unpublished data from the Louisiana and Mississippi state health departments document that MSM seek HIV testing/care services in neighboring jurisdictions.

Data were collected from March 2016 to August 2016. One study site (Hattiesburg) was removed from analyses due to protocol violations. Potential participants were considered eligible if they self-reported (1) African American or black race, (2) assigned male sex at birth, (3) being 18 years or older, (4) residence in one of the four study cities, and (5) oral or anal sex with another man within the 6 months prior to study enrollment. Participant recruitment was conducted via community-based sampling methods (eg, word-of-mouth, posted flyers). Enrollment was coordinated and facilitated by community-based organizations and AIDS-service organizations, specifically My Brother’s Keeper (Mississippi) and CrescentCare/The Movement Office and Priority Health Care (Louisiana).

Participants’ study involvement included an enrollment (baseline) visit and two follow-up visits (midpoint and completion) at the offices of the partnering community-based and AIDS-service organizations. The midpoint visit occurred approximately 7 days after the enrollment visit (usually on Day 7), and the completion visit occurred approximately 7 days after the midstudy survey visit (usually on Day 14). At the enrollment visit, participants were given a US $25 VISA gift card, a US $50 Shell gas card, and a bag of condoms and water-based lubricants as remuneration for their participation. At the midpoint visit, participants were given a US $75 VISA gift card and a bag of condoms and water-based lubricants. At the end of the completion visit, participants received a US $100 VISA gift card and a bag of condoms and water-based lubricants. While GPS tracking was conducted throughout the 2-week GPS protocol, a few participants came in for their visit after 14 days for various logistical reasons. All participants provided written informed consent prior to enrollment, and all protocols were approved by the Sterling Institutional Review Board. The secondary analyses reported here were determined to be exempt by the New York University School of Medicine Institutional Review Board.

### Global Positioning System Data Processing

Prior to distribution, we programmed the GPS device to log in in 30-second intervals. Consistent with our past GPS protocols [33], during the study orientation and baseline assessment, participants were instructed to place the small QStarz’s BT-Q1000XT GPS device on their belt (using the manufacturer-provided case), in their pocket, or to connect the GPS device in the provided case to an item on their person (such as to a key chain or backpack strap), and to complete a travel diary. Participants were asked to wear the GPS units at all times, except for sleeping, swimming, or showering. The travel diary asked the participant questions related to GPS protocol compliance: “Did you charge the GPS monitor today?” and “Did you carry the GPS monitor with you today?” This was meant to help the participant remember to charge the unit and carry it with him. The GPS device was given to participants in a large plastic zipper storage bag, which also contained a mini-USB charging cord for the GPS device, a USB wall adapter for charging, a manufacturer-provided GPS belt holder (if requested), a pamphlet containing background information on GPS, and the travel diary. GPS participant data were downloaded using the Qstarz proprietary software and stored as .gpx files. The GPS data were then cleaned using several scripts written in the Python programming language and ArcGIS models to eliminate duplicate data, GPS points likely caused by multipath reflectance, GPS data likely caused by timing errors, and isolated GPS data (Python Software Foundation, Python Language Reference, version 2.7) and ArcGIS version 10.2 (ESRI).

### Study Variables

#### Geosocial Networking App Use

##### Overall App Use

At the enrollment visit, we asked participants, “Have you EVER used a sexual networking mobile application or ‘app’ such as Grindr or Jack’d?” At the midpoint and completion visits, we asked, “In the past 7 days, have you used a mobile application or ‘app’ such as Grindr or Jack’d?” Response options were “Yes” and “No.”

##### Age of First App Use

At the enrollment visit, we asked participants, “How old were you when you started using sexual networking ‘apps’ such as Grindr, or Jack’d?” An open-ended response format was provided to the participant.

##### Specific Apps Used

At the enrollment visit, we asked, “Which of the following ‘apps’ do you have an account or profile?” We provided a list of apps, including Adam4Adam, BGC Live, Grindr, Hornet, Jack’d, and Scruff.

##### Reasons for App Use

At all three visits, we asked, “Which of the following describes your reason(s) for using these apps?” Response options included “I want to make new friends,” “I want to meet people to have sex/hook up with,” “I want to find someone to date,” “Bored; I want to kill time,” “I want to connect to the gay community,” and “I want to find people to drink/use drugs with.”

##### Self-Presentation on App Profiles

At the enrollment visit, we asked, “Which of the following describes the photo(s) on your profile?” Response options included “My profile picture shows my face,” “My profile picture shows my bare chest or abs,” “My profile picture shows my bare arms or biceps,” “My profile picture shows my bare back or shoulders,” “My profile picture shows my bare legs,” “My profile picture shows my bare buttocks (or ass),” “My profile picture shows my bare penis,” and “My profile picture does not show any part of my body.” Participants could select all that applied.

##### Logon Times and Duration

At the enrollment visit, we asked, “In the past 30 days, on average, how many times each day did you open or login to mobile ‘apps’ such as Grindr or Jack’d?” At the midpoint and completion visits, we asked, “In the past 7 days, on average, how many times each day did you open or login to a mobile ‘app’ such as Grindr or Jack’d?” An open-ended response format was provided for this item. At the enrollment visit, we also asked, “In the past 30 days, on average, how many hours and/or minutes did you spend each day on mobile ‘apps’ such as Grindr or Jack’d chatting or viewing photos or profiles?” At the midpoint and completion visits, we asked, “In the past 7 days, on average, how many hours and/or minutes did you spend each day on mobile ‘apps’ such as Grindr or Jack’d chatting or viewing photos or profiles?” An open-ended response format was provided for this item. Duration of app use reported in minutes was converted into hours for the analysis.

##### Messaging Behaviors

At the enrollment visit, we asked, “In the past 30 days, on average, how many messages did you *send* each day on mobile ‘apps’ such as Grindr or Jack’d?” At the midpoint and completion visits, we asked, “In the past 7 days, on average, how many messages did you *send* each day on these ‘apps’ such as Grindr or Jack’d?” An open-ended response format was provided to the participant. In addition, at the enrollment visit, we also asked, “In the past 30 days, on average, how many messages containing nude or suggestive photos did you *receive* each day on these ‘apps’ such as Grindr or Jack’d?” At the midpoint and completion visits, we asked, “In the past 7 days, on average, how many messages containing nude or suggestive photos did you *receive* each day on these ‘apps’ such as Grindr or Jack’d?” An open-ended response format was provided to the participant.

##### Sex Behaviors With App-Met Partners

At the enrollment visit, we asked, “In the past 30 days, how many men did you begin talking to on these mobile ‘apps,’ meet in person, and engage in anal sex as a TOP (where you put your penis in his anus/rectum)?” At the midpoint and completion visits, we asked, “In the past 7 days, how many men did you begin talking to on these apps, meet in person, and engage in anal sex as a TOP (where you put your penis in his anus/rectum)?” An open-ended response format was provided to the participant. Those who responded more than 0 to these questions, were asked, “Was a condom used each time from start to finish (ejaculation)?” at the enrollment, midpoint, and completion visits. Response options were “Yes” and “No.”

Additionally, at the enrollment visit, we asked, “In the past 30 days, how many men did you begin talking to on these mobile ‘apps,’ meet in person, and engage in anal sex as a BOTTOM (where he put his penis in your anus/rectum)?” At the midpoint and completion visits, we asked, “In the past 7 days, how many men did you begin talking to on these mobile ‘apps,’ meet in person, and engage in anal sex as a BOTTOM (where he put his penis in your anus/rectum)?” An open-ended response format was provided to the participant. Those who responded more than 0 to these questions were asked, “Was a condom used each time from start to finish (ejaculation)?” at the enrollment, midpoint, and completion visits. Response options were “Yes” and “No.”

##### Characteristics of Partners on Apps

We assessed multiple characteristics of the sexual partners met on these apps, including partner age and partner race/ethnicity. To examine partner age, at the enrollment visit, we asked, “In general, are the majority of your sexual partners met on these apps?” At midpoint and completion visits, we asked, “In general, in the past 7 days, were the majority of the men met on these apps?” Response options included “A lot older (>5 years),” “Slightly older (2-4 years),” “Approximately the same age,” and “Younger (≤3 years),” and “Unsure/Don't know.” “Did not have anal sex with any men met on ‘apps’ such as Grindr or Jack’d” was a response option at the midpoint and completion visits.

To assess race/ethnicity, at the enrollment midpoint and completion visits, we asked, “Are the majority of your sexual partners met on these apps Latino or Hispanic?” Response options included “Yes,” “No,” and “Unsure/Don’t Know.” In addition, at the enrollment, midpoint, and completion visits, we asked, “Are the majority of your sexual partners met on these apps?” Response options included “White,” “Black or African American,” “Asian Indian or Alaska Native,” “Asian”; “Native Hawaiian or Pacific Islander,” and “Unsure/Don’t Know.” To assess partner HIV status, at the enrollment, midpoint, and completion visits, participants were asked, “Are the majority of your sexual partners met on these apps?” Response options included “HIV Positive,” “HIV Negative,” and “I did not ask.”

#### Global Positioning System Activity Space Size Calculation

Using data from the 2-week period, GPS activity space buffers were created using ArcGIS version 10.3 (ESRI). There are various ways to define and conceptualize an activity space as well as various distance thresholds for these measurements [34]. In this study, we used a daily path area (a buffering zone drawn around the GPS tracks), which is a common method in behavioral geography research to understand where participants spend the majority of their time and exposure to environments [34]. We buffered all the processed and cleaned GPS points at four distances (400 m, 800 m, 1600 m, and 8000 m) and dissolved these buffers into a single feature to create an activity space for each participant at the various buffer distances. The activity space size was expressed in square kilometers.

#### Sociodemographic Variables

Sociodemographic data collected at the enrollment visit were analyzed. Age was categorized as 19-22, 23-25, and 26-29 years. Ethnicity included Latino/Hispanic or non-Latino/Hispanic. Sexual orientation included gay/homosexual, bisexual, straight/heterosexual, questioning, or “I do not identify with any of these.” Sexual attraction included attracted to males only, most attracted to males, equally attracted to males and females, attracted to females only, mostly attracted to females, or not sure. Sexual partners in past 6 months included men, women, transgender women, or transgender men. Education levels included less than a 12^th^ grade education, high school diploma or general educational development (GED), community college, trade school or vocational school, bachelor’s degree, or graduate degree or professional degree. Current student status was categorized as full-time or part-time. Employment status groups were defined as “working full-time,” “working part-time or occasionally,” “unemployed,” and “unable to work (disabled).” Annual household income was coded as <US $12,000; $12,000-$19,999; and $20,000+ categories. Current living situation included alone/by myself, roommate(s) or friend(s), parent(s) or other family member(s), partner or significant other (with or without children), and “I do not have a stable living situation/other.” Relationship status was coded as yes or no. Number of places lived in past 2 years was categorized 1, 2, and 3 or more. Vehicle ownership was coded as yes, no, or unknown. Study site included Jackson (Mississippi), Gulfport (Mississippi), or New Orleans (Louisiana). Participants reported their HIV status as negative, positive, or unknown.

## Results

### Statistical Analysis

Of the 75 participants who enrolled in the study (25 per site), 3 did not complete the study. One participant was arrested during the 2-week GPS protocol and spent time in jail; one participant was in a car accident and although the accident was not fatal, the participant withdrew from the study; and one participant withdrew from the study because of his demanding work schedule. At the midpoint visit, 3 participants (4%) did not complete the survey, yielding 72 who completed the survey. At the completion visit, 4 participants (5%) did not complete the survey, yielding 71 who completed the survey. For Table 1, data from 70 participants, which included both app users (n=53) and nonusers (n=17), was included in the analysis. We excluded 3 participants who did not respond to the question regarding the use of GSN apps. First, chi-square or Fisher’s exact test was used to compare the sociodemographic characteristics of individuals who reported using such apps with those of individuals who did not. Using GPS data, we calculated means and standard errors (SE) of the activity spaces (ie, 400 m, 800 m, 1600 m, and 8000 m buffer sizes). For the largest buffer size (8000 m) of the total activity spaces, we categorized data into quartiles based on the distribution of values. Student’s *t* tests were used to estimate for differences in mean activity spaces by GSN app user status. Table 2 reports the frequency with which each type of GSN app was used, the number of apps used, and the types of profile pictures among those who reported ever using GSN apps at the enrollment visit (53/70, 75.7%). In Table 3, we show the characteristics of app use (ie, age at first use of apps, reasons for using apps, and login times and durations), app-met sexual partners (ie, relative partner’s age, race/ethnicity, and HIV status), and app-related sexual behaviors (ie, engagement in anal sex and condom use) based on data obtained at baseline, the midpoint, and study completion. Those who reported ever using GSN apps at the enrollment visit (53/70, 75.7%) and those who reported using GSN apps in the past 7 days at midpoint visit (27/70, 38.5%) and completion visit (19/70, 27.1%) were included for the analyses. Descriptive statistics are presented as mean (standard deviation [SD]), range, and interquartile range (IQR) for continuous variables and sample size (n) with percentage for categorical variables. To compare between data obtained at midpoint and on completion, we combined these data. Five participants who reported *not* using GSN apps in the past 7 days at the midpoint visit reported using apps at the completion visit. Thus, a total of 32 participants were included in the analyses. McNemar’s test or the Wilcoxon signed-rank test was used as appropriate. Statistical significance was determined by *P*<.05. All statistical analyses were conducted with Stata version 14.0 (Stata Corp).

### Sample Sociodemographic Characteristics

The sociodemographic characteristics of the sample of YBMSM are presented in Table 1. Of the 70 participants, 76% (53/70) of the sample reported ever having used GSN apps and the mean age of this group was 24.7 years (SD 2.6). All participants self-identified their race as black, and approximately 8% of YBMSM using GSN apps reported Latino/Hispanic ethnicity. Approximately two-thirds of participants identified as homosexual: 68% (36/53) of app users and 65% (11/17) of nonusers. The majority of app users reported attraction to males: 91% (48/53) of app users versus 71% (12/17) of nonusers, *P*=.043. Among app users, 30% (16/53) reported being attracted to men only while 60% (32/53) reported being mostly attracted to men. Overall, there were no significant sociodemographic differences between app users and nonusers, except for sexual attraction. Additionally, 100% of the 53 app users reported that their sexual partners in the past 6 months were men. Approximately 6% (3/53) of app users had a high school education or less, and 45% (24/53) were enrolled in school. For 71% (37/53) of app users, the annual individual-level income was less than US $20,000. Just over 80% (43/53) of app users reported owning a vehicle, 38% (20/53) reported being in a committed relationship, and 28% (15/53) were HIV-positive.

### Spatial Mobility Among Sample

The activity space size of app users was usually larger than nonusers. Among app users, the overall average activity space (8000 m buffer) was 4057.19 km (SE 772.82). Among nonusers, the overall average activity space was 2281.02 km (SE 1149.61). For Quartile 3 (for the 8000 m buffer), there was a significant difference in GPS activity spaces between app using YBMSM compared to nonapp using YBMSM (2719.54 km^2^ vs 1855.68 km^2^, *P*=.011).

**Table 1:** Sample sociodemographics (N=70).

Demographic and socioeconomic variables			App users (n=53)	Nonusers (n=17)	*P* value^a^
	**Age, years, n (%)**				
		19-22	14 (26.4)	8 (47.1)	.182
		23-25	18 (34.0)	6 (35.3)	
		26-29	21 (39.6)	3 (17.7)	
	Latino/Hispanic ethnicity, n (%)		4 (7.6)	0 (0.0)	.566
	**Sexual orientation, n (%)**				
		Gay or homosexual	36 (67.9)	11 (64.7)	.117
		Bisexual	12 (22.6)	2 (11.8)	
		Straight or heterosexual	0 (0.0)	2 (11.8)	
		Questioning	1 (1.9)	1 (5.9)	
		Do not identify with any of these	4 (7.6)	1 (5.9)	
	**Sexual attraction, n (%)**				
		Attracted to males only	16 (30.2)	4 (23.5)	.043^b^
		Mostly attracted to males	32 (60.4)	8 (47.1)	
		Equally attracted to males and females	4 (7.6)	2 (11.8)	
		Attracted to females only	0 (0.0)	3 (17.7)	
		Not sure	1 (1.9)	0 (0.0)	
	**Sexual partners in past 6 months (missing=1), n (%)**				
		Men	53 (100.0)	13 (81.3)	N/A^c^
		Women	3 (5.7)	4 (25.0)	
		Transgender women	0 (0.0)	0 (0.0)	
		Transgender men	1 (1.9)	0 (0.0)	
	**Education, n (%)**				
		High school diploma or less	3 (5.7)	1 (5.9)	.851
		High school diploma or GED^d^	20 (37.7)	7 (41.2)	
		Community college/trade school/ vocational school	19 (35.9)	7 (41.2)	
		Bachelor’s degree	9 (17.0)	1 (5.9)	
		Graduate degree	2 (3.8)	1 (5.9)	
	**Currently enrolled in school, n (%)**				
		Yes	24 (45.3)	5 (29.4)	.248
		No	29 (54.7)	12 (70.6)	
	**Full-time student status (n=29), n (%)**				
		Full-time	16 (66.7)	2 (40.0)	.339
		Part-time	8 (33.3)	3 (60.0)	
	**Annual household income (missing=1), US$, n (%)**				
		<$12,000	18 (34.6)	11 (64.7)	.128
		$12,000-$19,999	19 (36.5)	3 (17.7)	
		$20,000+	15 (28.9)	3 (17.7)	
	**Current employment status, n (%)**				
		Full-time	24 (45.3)	9 (52.9)	.115
		Part-time or working occasionally	19 (35.9)	2 (11.8)	
		Unemployed	10 (18.9)	6 (35.3)	
	**Current living situation, n (%)**				
		Alone/by myself	12 (22.6)	7 (41.2)	.066
		Roommate(s)/friend(s)	20 (37.7)	3 (17.7)	
		Parent(s)/other family members	17 (32.1)	3 (17.7)	
		Partner/significant other	3 (5.7)	4 (23.5)	
		Other/unknown	1 (1.9)	0 (0.0)	
	**Committed relationship, n (%)**				
		Yes	20 (37.7)	9 (52.9)	.268
		No	33 (62.3)	8 (47.1)	
	**Number of places lived in past 2 years, n (%)**				
		1	21 (39.6)	6 (35.3)	.761
		2	24 (45.3)	7 (41.2)	
		≥3	8 (15.1)	4 (23.5)	
	**Vehicle ownership, n (%)**				
		Yes	43 (81.1)	13 (76.5)	.732
		No	10 (18.9)	4 (23.5)	
	**Study site, n (%)**				
		Jackson, MS	16 (30.2)	9 (52.9)	.235
		Gulfport, MS	18 (34.0)	4 (23.5)	
		New Orleans, LA	19 (35.9)	4 (23.5)	
	**HIV status (missing=1), n (%)**				
		HIV-infected	15 (28.9)	3 (17.7)	.638
		HIV-uninfected	35 (67.3)	14 (82.4)	
		Unknown/Do not know	2 (3.9)	0 (0.0)	
	**GPS activity space (missing=5), mean (SE)**				
		400 m	233.34 (35.23)	143.19 (53.01)	.195
		800 m	462.59 (78.19)	271.67 (116.97)	.215
		1600 m	893.67 (160.77)	509.19 (240.07)	.224
		8000 m	4057.19 (772.82)	2281.02 (1149.61)	.243
	**Quartile of GPS activity space (8000 m), mean (SE)**				
		Quartile 1	606.77 (57.13)	650.86 (58.81)	.657
		Quartile 2	1016.69 (40.81)	1065.62 (72.93)	.554
		Quartile 3	2719.54 (157.83)	1855.68 (109.70)	.011^b^
		Quartile 4	9252.10 (1742.20)	19426.56 (—)	—

^a^Chi-square test, Fisher’s exact test or two-sample *t* test was conducted as appropriate.

^b^Statistically significant.

^c^N/A: not applicable. Multiple response (categories are not mutually exclusive).

^d^GED: General Education Diploma.

**Table 2:** Geosocial networking app use^a^ among MSM (N=53^b^).

Geosocial app use^c^	n (%)
Adam4Adam	22 (41.5)
BCG Live	15 (28.3)
Bender	0 (0.0)
Boy Ahoy	3 (5.7)
Distinc.tt	0 (0.0)
DowneLink	2 (3.8)
Grindr	19 (35.9)
GROWLr	3 (5.7)
Guy Spy	0 (0.0)
Hornet	3 (5.7)
Hunters BBS	0 (0.0)
Jack’d	37 (69.8)
Maleforce	0 (0.0)
Planet Romeo	3 (5.7)
Scruff	2 (3.8)
Skout	2 (3.8)
Tinder	3 (5.7)
U2nite	0 (0.0)
U4Bear	1 (1.9)
VGL	0 (0.0)
Other	7 (13.2)
Instagram	39 (73.6)
Kik	27 (50.9)
SnapChat	39 (73.6)

^a^Assessed in the enrollment visit.

^b^Those who reported ever using GSN apps at the enrollment visit.

^c^Multiple response (categories are not mutually exclusive).

### Geosocial Networking App Use

Table 2 shows GSN app use among respondents who reported having ever used GSN apps at the enrollment visit. Jack’d was a popular GSN app (37/53, 69.8%), followed by Adam4Adam (22/53, 41.5%), and Grindr (19/53, 35.9%). Nearly three-quarters of the sample reported using Instagram and Snapchat apps. The number of GSN apps used varied among the sample, with values ranging from 0-13. The mean and median number of apps used were 4.3 (SD 2.66) and 4.0 (range 0-13), respectively. Most of the sample (47/53, 88.7%) reported using 2 or more apps. In terms of the body content appearing on the profile pictures, most participants displayed their face (35/52, 67.3%) followed by their bare chest or abs (10/52, 19.2%), and bare penis (4/52, 7.7%), whereas fewer displayed their bare legs (2/52, 3.9%) or bare buttocks (or ass; 2/52, 3.9%).

Table 3 shows app use across the three study time points. The mean age at initiation of app(s) use was 20.1 years (SD 2.8), with values ranging from 13-26 years. Among 53 participants at baseline, an average of 8.2 (SD 16.8) apps were logged onto or opened each day during the past 30 days, and an average of 2.1 (SD 3.1) hours was spent each day in the past 30 days chatting or viewing photos/profiles on the apps. Two-thirds of the sample reported using the apps to “kill time,” just over half reported using the apps to make new friends, and approximately a third reported using the apps to connect to the gay community or to find someone to date. Only 9 participants reported using the apps to meet people to have sex/hook up with.

**Table 3:** Messaging behaviors across three time periods among app-using men who have sex with men.

Messaging behaviors		Baseline (n=53)	Midpoint (n=27)	Completion (n=19)	*P* value^a^
Age when started using app, mean (SD), range, interquartile range (IQR)		20.06 (2.78), 13-26, 18-22	N/A	N/A	—
**Reason for using these apps^b^, n (%)**					
	I want to make new friends	29 (54.7)	18 (66.7)	12 (63.2)	—^c^
	I want to meet people to have sex/hook up with	9 (17.0)	4 (14.8)	4 (21.1)	.564
	I want to find someone to date	16 (30.2)	7 (25.9)	5 (26.3)	.564
	Bored; I want to kill time	35 (66.0)	25 (92.6)	12 (63.2)	.317
	I want to connect to the gay community	17 (32.1)	9 (33.3)	8 (42.1)	.999
	I want to find people to drink/use drugs with	0 (0.0)	0 (0.0)	0 (0.0)	—^c^
Number of times (on average) opened or logged on to apps each day in past 30 days, mean (SD), range, IQR		8.21 (16.79), 0-100, 0-9	N/A	N/A	—
Number of times (on average) you opened or logged on to apps in past 7 days, mean (SD), range, IQR		N/A	9.26 (18.73), 1-100, 3-9	6.16 (7.03), 0-30, 2-10	.184
Hours (on average) spent each day on apps chatting or viewing photos or profiles in past 30 days, mean (SD), range, IQR		2.06 (3.07), 0-12.50, 0.08-2.50	N/A	N/A	—
Hours (on average) spent each day on apps chatting or viewing photos or profiles in past 7 days, mean (SD), range, IQR		N/A	2.68 (3.57), 0.08-15.00, 0.33-3.75	3.35 (5.73), 0-23, 0.5-3.0	.875
Number of messages (on average) *sent* each day on apps in past 30 days (missing=1), mean (SD), range, IQR		10.06 (27.84), 0-200, >0-10	N/A	N/A	—
Number of messages (on average) *sent* each day on apps in past 7 days, mean (SD), range, IQR		N/A	8.44 (10.17), 0-50, 3-10	5.74 (7.19), 0-30, 3-5	.055
Number of messages (on average) *received* each day on apps in past 30 days, mean (SD), range, IQR		27.02 (55.64), 0-300, 2-25	N/A	N/A	—
Number of messages (on average) *received* each day on apps in past 7 days, mean (SD), range, IQR		N/A	14.33 (14.59), 3-70, 5-20	9.89 (9.51), 0-30, 3-10	.079

^a^McNemar’s test or Wilcoxon sign-rank test was conducted as appropriate to compare characteristics between midpoint and completion survey.

^b^Multiple response (categories are not mutually exclusive).

^c^Could not estimate because of sparse cells.

**Table 4:** Sex behaviors with app-met partners across three time periods among app-using men who have sex with men.

Sex behaviors with app-met partners	Baseline (n=53)	Midpoint (n=27)	Completion (n=19)	*P* value^a^
Number of explicit or x-rated messages (on average) *received* on mobile apps, mean (SD), range, interquartile range (IQR)	18.43 (35.65), 0-200, 0-15	N/A^b^	N/A	—
Number of messages (on average) containing nude or suggestive photos you *sent* each day on apps in the past 30 days, mean (SD), range, IQR	3.13 (6.21), 0-30, 0-3	N/A	N/A	—
Number of messages (on average) containing nude or suggestive photos you *sent* each day on apps in the past 7 days, mean (SD), range, IQR	N/A	1.81 (3.29), 0-15, 0-3	1.00 (1.56), 0-5, 0-2	.276
Number of messages (on average) containing nude or suggestive photos you *received* each day on apps in the past 30 days, mean (SD), range, IQR	33.15 (139.25), 0-1000, 0-15	N/A	N/A	—
Number of messages (on average) containing nude or suggestive photos you *received* each day on apps in the past 7 days, mean (SD), range, IQR	N/A	4.81 (6.88), 0-25, 0-5	3.58 (4.73), 0-20, 0-5	.046^c^
Number of men you began talking on the these apps, met in person, and engaged in anal sex as a TOP in the past 30 days, mean (SD), range, IQR	1.19 (3.57), 0-25, 0-1	N/A	N/A	—
Condom used each time from start to finish (ejaculation), n (%)	12/17 (70.6)	N/A	N/A	—
Number of men you began talking on the these apps, met in person, and engaged in anal sex as a TOP in the past 7 days, mean (SD), range, IQR	N/A	0.30 (0.72), 0-3, 0-0	0.26 (0.56), 0-2, 0-0	.103
Condom used each time from start to finish (ejaculation), n (%)	N/A	4/5 (80.0)	3/4 (75.0)	.317
Number of men you began talking on these apps, met in person, and engaged in anal sex as a BOTTOM in the past 30 days, mean (SD), range, IQR	0.58 (1.31), 0-7, 0-1	N/A	N/A	—
Condom used each time from start to finish (ejaculation), n (%)	13/15 (86.7)	N/A	N/A	—
Number of men you began talking on these apps, met in person, and engaged in anal sex as a BOTTOM in the past 7 days, mean (SD), range, IQR	N/A	0.04 (0.19), 0-1, 0-0	0.05 (0.23), 0-1, 0-0	—^d^
Condom used each time from start to finish (ejaculation), n (%)	N/A	0/1 (0)	1/1 (100.0)	—^d^

^a^McNemar’s test or Wilcoxon sign-rank test was conducted as appropriate to compare characteristics between midpoint and completion survey.

^b^N/A: not applicable.

^c^Statistically significant.

^d^Could not estimate because of sparse cells.

### Sexual Behaviors With and Characteristics of App-Met Partners

Table 4 shows the sexual behaviors that participants engaged in with app-met partners. At baseline, participants had 1.2 app-met male partners (SD 3.6) during the past 30 days who they engaged in anal sex with as a “Top”; 70.6% (12/17) of them used condoms throughout the sexual encounter. According to the data collected at the midpoint, the range of male partners from who they engaged in anal sex as the Top were 0-3; participants reported from 0-2 at the completion visit. At baseline, respondents had 0.6 app-met male partners engaged in anal sex as the “Bottom” in the past 30 days, and more than three-quarters (13/15, 86.7%) of them used condoms throughout the sexual encounter. The average numbers of outgoing and incoming app-delivered messages containing nude/suggestive photos in the past 30 days were 3.1 and 33.2, respectively.

Table 5 shows the characteristics of app-met partners. The majority of the baseline sample reported meeting partners approximately the same age or slightly older (43/51, 84.3%) than themselves during the past 30 days. In the midpoint and final surveys, approximately 35% (10/27 and 7/19) reported that they met partners approximately the same age. The vast majority of participants reported meeting black partners across all three time points. At baseline, over two-thirds (36/53, 67.9%) reported that the HIV status of their app-met partners was negative, and 26.4% (14/53) reported that they did not know their partner’s HIV status. Most participants also reported their app-met partners’ HIV status as negative or unknown at the midpoint and completion visits.

**Table 5:** App-met partner characteristics across three time periods among app-using men who have sex with men.

App-met partner characteristics		Baseline (n=53), n (%)	Midpoint (n=27), n (%)	Completion (n=19), n (%)	*P* value^a^
**The majority of your sexual partners met on these apps in the past 30 days (missing=2)**					
	A lot older (>5 years)	4 (7.8)	N/A^b^	N/A	–
	Slightly older (2-4 years)	21 (41.2)	N/A	N/A	–
	Approximately the same age	22 (43.1)	N/A	N/A	–
	Younger (≤3 years)	4 (7.8)	N/A	N/A	–
	Don’t know/not sure	0 (0.0)	N/A	N/A	–
**The majority of the men met on these apps in the past 7 days**					
	A lot older (>5 years)	N/A	1 (3.7)	1 (5.3)	–^c^
	Slightly older (2-4 years)	N/A	7 (25.9)	3 (15.8)	.564
	Approximately the same age	N/A	10 (37.0)	7 (36.8)	.999
	Younger (≤3 years)	N/A	1 (3.7)	1 (5.3)	–^c^
	Did not have anal sex with any men met	N/A	8 (29.6)	7 (36.8)	.564
**The majority of your sexual partners met on these apps: Latino or Hispanic**					
	Yes	3 (5.7)	1 (3.7)	0 (0.0)	–^c^
	No	49 (92.5)	18 (66.7)	12 (63.2)	–
	Missing	1 (1.9)	8 (29.6)	7 (36.8)	–
**The majority of your sexual partners met on these apps**					
	White	1 (1.9)	1 (3.7)	0 (0.0)	–^c^
	Black or African American	51 (96.2)	18 (66.7)	12 (63.2)	–
	Asian Indian or Alaska Native	0 (0.0)	0 (0.0)	0 (0.0)	–
	Asian	0 (0.0)	0 (0.0)	0 (0.0)	–
	Native Hawaiian or Pacific Islander	0 (0.0)	0 (0.0)	0 (0.0)	–
	Missing	1 (1.9)	8 (29.6)	7(36.8)	–
**The majority of your sexual partners met on these apps**					
	HIV positive	2 (3.8)	0 (0.0)	0 (0.0)	.317
	HIV negative	36 (67.9)	9 (33.3)	7 (36.8)	–
	I did not ask	14 (26.4)	10 (37.0)	5 (26.3)	–
	Missing	1 (1.9)	8 (29.6)	7 (36.8)	–

^a^McNemar’s test or Wilcoxon sign-rank test was conducted as appropriate to compare characteristics between midpoint and completion survey.

^b^N/A: not applicable.

^c^Could not estimate because of sparse cells.

## Discussion

### Principal Findings

To our knowledge, this is the first study to examine GSN app use patterns among YBMSM in the southern United States, specifically the Deep South. The majority (76%) of the sample reported using GSN apps and reported using multiple apps. Jack’d was the most commonly used GSN app. Interestingly, few participants (17%) in our sample reported using Grindr, which is in contrast to previous research with racially diverse MSM samples [9]. To date, YBMSM have been underrepresented in MSM studies recruited from GSN apps, perhaps because many of these samples have been recruited from Grindr [18,19,21]. Therefore, our study is an important contribution to the literature.

The mean age at initiation of app use was 20.1 years with the youngest reported age being 13 years old, which reinforces the idea that GSN apps are a relevant virtual venue to reach YBMSM for HIV prevention and treatment messaging. Despite research showing that MSM commonly meet sexual partners on GSN apps, over 60% (35/53) of the sample reported that their primary reason for using these apps was to “kill time.” These findings suggest GSN apps could be useful for sending HIV prevention-related messages through advertisements [35] or through direct messaging [36]. Similar to other studies demonstrating high levels of assortative mixing based on race/ethnicity in the networks of BMSM [25,37], the vast majority of participants reported meeting black sexual partners on these apps.

Interestingly, less than half of app users reported being in a committed relationship. The majority of the baseline sample reported meeting partners approximately the same age or slightly older than themselves. This is significant because age discordance, particularly meeting older partners, is associated with increased HIV risk [25,38-40]. Over two-thirds of participants at the enrollment visit reported that the HIV status of their app-met partners was negative. Almost one-third of participants did not know the status of their partner. If BMSM are meeting and having CAI with people they think are HIV negative (because they self-reported their HIV status as negative or unknown) on an app, this could lead to increased HIV acquisition. This finding of not knowing one’s partners’ HIV status points to the need for further communication in sexual dyads about HIV risk reduction practices [41]. Moreover, these findings illustrate the need for encouraging the disclosure of HIV status and decreasing stigma for HIV-positive users on GSN apps. Interestingly, in terms of the body content appearing on the profile pictures, most participants (over two-thirds) displayed their face. Compared to nonusers, a majority of app users reported attraction to males.

Additionally, because we found inconsistent condom use with app-met partners, on GSN apps there should be increased efforts promoting condom use, HIV testing, treatment as prevention for those living with HIV, and PrEP for those who are HIV-negative. This is especially important in the context of the low number of reported sexual partners, which is consistent with the BMSM paradox and previous research showing few sexual partners among BMSM [25]. The intersection of inconsistent condom use and a higher HIV prevalence among older BMSM (many of whom have unknown HIV statuses) presents a critical need for interventions among young BMSM in the Deep South.

Compared to previous research among app-using MSM in the Deep South, our study reported similar reasons for and patterns of GSN app use. A prior study (where MSM from a single city in the Deep South were recruited from a single popular app) found that over one-third used mobile apps to meet new sexual partners and about one-fifth used them to “kill time” when bored [9]. In that study, MSM participants had current accounts on 3.1 mobile apps per day, with Grindr being the most common (100%), followed by Scruff (52.5%), and Jack’d (45.7%). Each day, men reported opening these mobile apps 8.4 times and spending 1.3 hours on these mobile apps. However, two notable differences between the current study and the aforementioned study are the respondents’ race/ethnicity and the apps most commonly used. Our study was composed of 100% BMSM while the other was 19% BMSM; Jack’d was the most popular GSN app in the current study, while Grindr was the most common app in the other study.

### Future Research

Further research can contribute to a better understanding of the role of GSN apps in the sexual lives of YBMSM and the way that app use behaviors may contribute to the increased risk of HIV infection among this population. Future research among YBMSM in the Deep South should be conducted through population-based samples and larger samples to further understand the prevalence and common patterns of GSN app use. Given that YBMSM who use GSN apps represent a diverse population in terms of their motivations and patterns of use as well as their sexual behaviors with app-met partners, future research should apply novel techniques, such as latent class analysis [42,43] to further understand the associations of various GSN app use profiles with risk for HIV infection. Latent class analysis is a useful tool to explore conditional probabilities of co-occurring behaviors within distinct classes and has been used to identify sexual risk profiles among black MSM [44]. Additionally, future investigations should include longitudinal designs to understand potential changes in GSN app use and associated changes in sexual risks, since GSN app use could be contextual and change as individual life circumstances change (eg, changes in partner type). Other research has found that sexual risks among BMSM vary with differing life phases and contexts of sexual relationships [45]. Future research should assess the HIV status that participants include in their app profile(s) and should also examine homophily in partner selection, such as HIV status homophily, as this may drive partner selections. Future research could also ask participants about their app-met partners’ PrEP use (if HIV-negative) or antiretroviral therapy use (if HIV-positive), as these factors may have an influence on app use or meeting partners. Additionally, given that GSN apps use GPS technology to connect nearby users, future research employing GPS tracking may be of use in understanding how GSN app use links MSM across geographies. GPS tracking could also illuminate how geography plays a role in transitioning user interactions from virtual app-based conversations to physical meetings for sexual encounters. It would also be helpful to know what influences certain patterns of app use and whether these influences differ from those in other geographic regions. This is especially important in the Deep South given its high rates of HIV infection; perhaps in this region there are specific geographical features that increase risk of HIV acquisition. Furthermore, as previously mentioned, it would be helpful to know what app use behaviors leads to certain health behaviors (including sexual health behaviors) as well as how app-based interventions could modify these behaviors. Future research studying GSN app use among YBMSM or using advertising capabilities on GSN apps could recruit YBMSM into HIV prevention and treatment programs. For example, research could examine app-specific promotions of PrEP use among YBMSM.

### Strengths and Limitations

Our study’s small sample was recruited through community-based methods via local organizations, including AIDS-service organizations. Subsequently, the sample may be subject to selection bias and therefore may not be representative of the wider population of BMSM in the Deep South. However, our study presents a strength over previous app-based research with MSM, which has traditionally been limited to white participants in a single geographic region, often recruited from apps. Our sample represents a smaller yet broader cross-section of BMSM who use GSN apps across three cities in the Deep South. More importantly, our study prompts future research into GSN app use among this subpopulation and highlights the need for more focused prevention efforts. Additionally, given the small sample size, we might not be adequately powered to compare participants who use GSN apps to those who do not. All data were collected via self-report, introducing potential for social desirability bias (ie, underreporting instances of CAI) and recall bias. In addition, we assessed behaviors using different time periods across the three waves of data collection, making comparison across waves difficult. While behaviors may differ depending on the app and many participants reported using multiple apps, we did not analyze behaviors by individual apps. Nonetheless, our findings represent a novel characterization of GSN app use among YBMSM in the Deep South—a topic and population warranting further study given the high rates of HIV diagnoses

### Conclusions

Among our sample of YBMSM in the Deep South, GSN app use for the purpose of meeting sexual partners was common, with a diverse range of app use behaviors being reported. Further research should characterize the association between GSN app use and engagement in sexual behaviors that increase risk for HIV acquisition and transmission. In addition, HIV prevention interventions for YBMSM who use GSN apps should be developed and evaluated. It should be noted that GSN apps themselves present important platforms for such interventions.

## Abbreviations

BMSM: black men who have sex with men
CAI: condomless anal intercourse
GSN: geosocial networking
GPS: global positioning system
HIV: human immunodeficiency virus
IQR: interquartile range
MSM: men who have sex with men
PrEP: pre-exposure prophylaxis
YBMSM: young black men who have sex with men
